# Supplementary material for: Psychometric Assessment of the Communication Skills Scale Among Peruvian Nurses and Factors Associated with Job Insecurity
Source: Healthcare (Basel). 2024 Dec 22;12(24):2582. doi: 10.3390/healthcare12242582 (PMC11728042; doi:10.3390/healthcare12242582)
Supplement: Supplementary file 1 [file healthcare-12-02582-s001.zip › Supplementary Material S1.pdf]

ITEMS BEFORE AND AFTER THE CULTURAL ADAPTATION PROCESS OF THE COMMUNICATION SKILLS SCALE APPLIED TO PERUVIAN NURSES

Item No.	Item of the Original scale	Modified Item
1	*Respeto el derecho de los pacientes a expresarse libremente* [I respect the right of patients to express themselves freely].	**Unchanged**
2	*Exploro las emociones de mis pacientes* [I explore my patients' emotions].	**Unchanged**
3	*Respeto la autonomía y libertad de los pacientes* [I respect patients' autonomy and freedom].	**Unchanged**
4	*Cuando el paciente me habla muestro interés mediante gestos corporales (asintiendo con la cabeza, contacto ocular, sonrisas…)* [When patients talk to me, I show interest through body gestures (nodding, eye contact, smiles...)].	*Cuando* ***mi*** *paciente me habla muestro interés mediante gestos corporales (asintiendo con la cabeza, contacto* ***visual,*** *sonrisas****, tocar el hombro o la mano)*** [When **my** patients talk to me, I show interest through body gestures (nodding, eye contact, smiles, **touching their shoulder or hand**...)].
5	*Proporciono información a los pacientes (siempre que mi competencia profesional me lo permita) sobre aquello que les preocupa* [I provide information to patients (as far as my professional competence allows me to do so) about their concerns].	*Proporciono información a los pacientes****/familiares, sobre los cuidados de salud*** *(con base a mi competencia profesional* ***y norma institucional****)* [I provide information to patients**/relatives on health care** (**based on** my professional competence **and institutional rules**)].
6	*Escucho a los pacientes sin prejuicios, independientemente de su aspecto físico, modales, forma de expresión…* [I listen to patients without prejudice, regardless of their physical appearance, manners, way of expression...].	**Unchanged**
7	*Expreso claramente mis opiniones y deseos a los pacientes* [I clearly express my opinions and wishes to patients].	**Unchanged**
8	*Cuando doy información, uso silencios para que el paciente asimile lo que le estoy diciendo* [When I give information, I use silences so that patients assimilate what I am saying].	*Cuando* ***brindo*** *información, uso silencios para que* ***mi*** *paciente* ***logre comprender mejor*** *lo que le estoy diciendo* [When I **provide** information, I use silences so that **my** patients **can better understand** what I am saying].
9	*Cuando doy información a los pacientes, lo hago en términos comprensibles* [When I give information to patients, I do so in understandable terms].	*Cuando* ***brindo*** *información a* ***mis*** *pacientes, lo* ***realizo*** *en términos* ***sencillos y con ejemplos*** [When I **provide** information to **my** patients, I do so in **simple** terms **and using examples**].
10	*Cuando un paciente hace algo que no me parece bien, le expreso mi desacuerdo o molestia* [When patients do something that does not seem right	*Cuando un paciente* ***realiza*** *algo que no me parece bien****,*** *le expreso mi desacuerdo* ***con amabilidad*** [When patients do something that

	to me, I express them my disagreement or discomfort].	does not seem right to me, I **kindly** express them my disagreement].
**11**	*Dedico tiempo para escuchar y tratar de comprender las necesidades de los pacientes* [I take time to listen and try to understand patients' needs].	*Dedico tiempo para **observar** y **relacionarme con mis** pacientes **a fin de valorar** sus necesidades* [I take time to **observe** and **interact with my** patients **in order to assess** their needs].
**12**	*Trato de comprender los sentimientos de mi paciente* [I try to understand my patients' feelings].	*Trato de comprender los sentimientos **y emociones** de mis pacientes* [I try to understand my patients' feelings **and emotions**].
**13**	*Cuando me relaciono con los pacientes, expreso mis comentarios de una manera clara y firme* [When interacting with patients, I express my comments clearly and firmly].	*Cuando me relaciono con **mis** pacientes, **les expreso que el cuidado de la salud es una responsabilidad compartida*** [When interacting with **my** patients, I express **to them that health care is a shared responsibility**].
**14**	*Considero que el paciente tiene derecho a recibir información sanitaria* [I consider that patients have the right to receive health information].	**Unchanged**
**15**	*Siento que respeto las necesidades de los pacientes* [I feel that I respect patients' needs].	*Siento que respeto las necesidades de los pacientes **sus creencias, valores y costumbres*** [I feel that I respect patients' needs, **beliefs, values, and traditions**].
**16**	*Me resulta difícil realizar peticiones a los pacientes* [I find it difficult to make requests to patients].	**Unchanged**
**17**	*Me aseguro que los pacientes han comprendido la información proporcionada* [I make sure that patients have understood the information provided].	*Me aseguro que **mis** pacientes **hayan** comprendido la información proporcionada* [I make sure that **my** patients **understood** the information provided].
**18**	*Me resulta difícil pedir información a los pacientes* [I find it difficult to ask patients for information].	**Unchanged**
**19***		Escucho a mis pacientes sin prejuicios, independientemente de mis creencias [I listen to my patients without prejudices, regardless of my beliefs)]
**20***		Al interactuar con los pacientes/familiares en situación de crisis, busco regular emociones y resolver conflictos [When interacting with patients/relatives in crisis, I try to regulate emotions and resolve conflicts)].

Note: items 19 and item 20 were added after the cultural adaptation process.
