# Supplementary material for: Psychometric Assessment of the Communication Skills Scale Among Peruvian Nurses and Factors Associated with Job Insecurity
Source: Healthcare (Basel). 2024 Dec 22;12(24):2582. doi: 10.3390/healthcare12242582 (PMC11728042; doi:10.3390/healthcare12242582)
Supplement: Supplementary file 1 [file healthcare-12-02582-s001.zip › Supplementary Material S2.pdf]

SUPPLEMENTARY MATERIAL 2

THE COMMUNICATION SKILLS SCALE FOR MEASURING NURSING PROFESSIONALS' COMMUNICATION SKILLS

Dear nursing professional, I am Gabriela Samillán, M.Sc., professor at the Universidad Nacional Mayor de San Marcos. Considering that communication skills are essential in the nursing dynamics and that they need constant self-assessment and improvement to achieve higher levels of job satisfaction, I am requesting nurses to anonymously complete this Communication Skills Scale questionnaire. Please answer the following items as accurate as possible so that we can validate the scale afterwards.

Informed consent (Mark only one box):

☐ I agree
☐ I do not agree

General Information

1. Sex:

   ☐ Male ☐ Female

2. Marital Status:

   ☐ Single ☐ Married / Cohabitating ☐ Widow
   ☐ Separated / Divorced

3. Age:
   ________ years old

4. Where do you primarily work at the moment?

   ☐ Hospital ☐ Healthcare Center / Facility ☐ Polyclinic ☐ Clinic
   ☐ Private Practice ☐ Other: ______________________________

5. How many years of experience do you have?

   ________ years

6. What type of employment contract do you have? (Mark only one box)

   ☐ Open-ended or tenured-employee contract
   ☐ Temporary administrative service contract in the public sector
   ☐ Permanent contract (DS 728)
   ☐ Fixed-term contract (subject to modality)
   ☐ Professional fees
   ☐ Other: ______________________________

7. Overall, how would you rate your level of satisfaction with your job or place of work?

   ☐ Satisfied

☐ Neither satisfied nor dissatisfied
☐ Dissatisfied

8. What is your department of residence?

   ____

9. Are you working remotely?

   ☐ Yes ☐ No

10. Did you become infected with COVID-19 during the pandemic?

    ☐ Yes ☐ No

11. Have you provided care to people infected with COVID-19 in the last three months?

    ☐ Yes ☐ No

COMMUNICATION SKILLS QUESTIONNAIRE

Study validating the scale: https://doi.org/10.23938/ASSN.0745

SPECIFIC DATA:

Directions

Below you will find 18 items with various response options. Please read each question carefully and answer them by selecting the option that best represents you. Note that your responses should reflect the frequency of your in-person communication with your patients.

Item No.	Items or Indicators	*Casi nunca* [Almost Never]	*Raras veces* [Rarely]	*A veces* [Sometimes]	*Siempre* [Always]	*Muchas veces* [Frequently]	*Muchísimas veces* [Very Frequently]
		1	2	3	4	5	6
1	*Respeto el derecho de los pacientes a expresarse libremente* [I respect the right of patients to express themselves freely].						
2	*Exploro las emociones de mis pacientes* [I explore my patients' emotions].						
3	*Respeto la autonomía y libertad de los pacientes* [I respect patients' autonomy and freedom].						
4	*Cuando el paciente me habla muestro interés mediante gestos corporales (asintiendo con la cabeza, contacto ocular, sonrisas…)* [When patients talk to me, I show interest through body gestures (nodding, eye contact, smiles...)].						
5	*Proporciono información a los pacientes (siempre que mi competencia profesional me lo permita) sobre aquello que les preocupa* [I provide information to patients (as far as my professional competence allows me to do so) about their concerns].						
6	*Escucho a los pacientes sin prejuicios, independientemente de su aspecto físico, modales, forma de expresión…* [I listen to patients without prejudice, regardless of their physical appearance, manners, way of expression...].						
7	*Expreso claramente mis opiniones y deseos a los pacientes* [I clearly express my opinions and wishes to patients].						
8	*Cuando doy información, uso silencios para que el paciente asimile lo que le estoy diciendo* [When I give information, I use silences so that patients assimilate what I am saying].						
9	Cuando doy información a los pacientes, lo hago en términos comprensibles [When I give information to patients, I do so in understandable terms].						
10	*Cuando un paciente hace algo que no me parece bien, le expreso mi desacuerdo o*						

	molestia [When patients do something that does not seem right to me, I express them my disagreement or discomfort].						
**11**	*Dedico tiempo para escuchar y tratar de comprender las necesidades de los pacientes* [I take time to listen and try to understand patients' needs].						
**12**	*Trato de comprender los sentimientos de mi paciente* [I try to understand my patients' feelings].						
**13**	*Cuando me relaciono con los pacientes, expreso mis comentarios de una manera clara y firme* [When interacting with patients, I express my comments clearly and firmly].						
**14**	*Considero que el paciente tiene derecho a recibir información sanitaria* [I consider that patients have the right to receive health information].						
**15**	*Siento que respeto las necesidades de los pacientes* [I feel that I respect patients' needs].						
**17**	*Me aseguro que los pacientes han comprendido la información proporcionada* [I make sure that patients have understood the information provided].						
**19**	Escucho a mis pacientes sin prejuicios, independientemente de mis creencias [I listen to my patients without prejudices, regardless of my beliefs)]						
**20**	Al interactuar con los pacientes/familiares en situación de crisis, busco regular emociones y resolver conflictos [When interacting with patients/relatives in crisis, I try to regulate emotions and resolve conflicts)].						
